# Changes in Medical Schools

**Published:** 1853-07

**Authors:** 


### CHANGES IN MEDICAL SCHOOLS.

The vacancy in the University of Buffalo, caused by the resignation of Professor Flint, has been filled by the election of Thomas F. Rochester, M. D., of the city of New York, to the chair of the Principles and Practice of Medicine and of Clinical Medicine. Dr. Rochester is represented to be a young man of talents, learning and industry, "an easy and spirited lecturer, and eminently a gentleman in his feelings and manners." We congratulate our friends at Buffalo on their success in procuring the services of so worthy an incumbent of that important chair.

In the Kentucky School of Medicine and Surgery Dr. Foree has resigned the chair of Materia Medica, and is succeeded by Robert J. Breckinridge, M. D., of this city. Dr. Breckinridge is known to many of our readers as a lecturer and writer. He is a young man of the finest talents, and is destined, we have no doubt, to take a high rank in his profession as a teacher and practitioner.

We learn that Professors Cobb and Baxley have resigned their chairs in the Medical College of Ohio, but have not heard who succeed them.

